# Miscellaneous Items

**Published:** 1854-12

**Authors:** 


### MISCELLANEOUS ITEMS.

LYING IN BED WITH THE HEAD HIGH.—It is often a question among people who are unaquainted with the anatomy and physiology of man, whether lying with the head exalted, or even with the body, was most wholesome. Most, consulting their own ease on this point, argue in favor of that which they prefer. Now, although many delight in bolstering up their heads at night, and sleep soundly, without injury, yet we declare it to be a dangerous habit. The vessels through which the blood passes from the heart to the head, are always lessened in their cavities when the head is resting in bed higher than the body, therefore in all diseases attended with fever, the head should be pretty nearly on a level with the body; and people ought to accustom themselves to sleep thus to avoid danger.—*Medical Journal.*

---

ELBOW PRECAUTION.—A gentleman from Morrisania, had his elbow broken lately, while leaning his arm outside the car on the Harlem railroad. A similar accident befel another gentleman on one of the city cars not long ago. The proper place for the elbow in all vehicles, as at the table and public assemblies, is beside the owner's person; a knowledge and remembrance of this may save some reader of the Journal the price of twenty years subscription. Three-fourths of those who ride in the omnibusses and city cars, sit with the elbows projecting outside: about a year ago, a gentleman had his arm shattered in that position, at the corner of Centre and Canal streets. Another and more fatal form of accident is of almost weekly occurrence, which the delay of three seconds would infallibly prevent. I allude to jumping on and off the city horse cars while in motion, *from the forward platform.* If a man's time is of such immense importance that he must save a single minute at the cost of risking a limb or life, a delay of three seconds will give him a chance of jumping on the rear platform, while the car is passing; if he misses his mark, he has only to get up out of the mud and give his washerwoman an extra shilling or two, while the same "miss" at the forward platform would have thrown him under the wheels and cost him his life, as has just been the case with a city editor, whose very last editorial was said to have been on cautioning his readers against that very practice.

How much Sugar do we Eat!—A western paper states, that 1853, there were consumed in this country about 705,000,000 pounds of cane sugar, and 27,000,000 pounds of maple sugar. This gives more than 24 pounds of cane sugar and one pound of maple sugar to every man, woman and child. This does not include molasses or honey. If this sugar were put into barrels holding 200 pounds, and each barrel occupied the space of three square feet only, it would require 336 acres of land for it to stand upon. The barrels, if placed in a row, would reach 220 miles. If the sugar was put up in paper packages of five pounds each, it would require 146,400,000 sheets of wrapping-paper; and if only a yard of string was used to each package, there would be required 439,200,000 feet, or 83,000 miles of string—more than three times enough to go round the world. If every retail clerk sold a hundred pounds of sugar each day, it would require nearly 25,000 clerks to sell it all in a year. If the dealers, wholesale and retail together, made a profit of only two cents a pound on this sugar, these profits would amount to nearly $15,000,000.

---

How much they Eat in New-York.—During the three months ending the 1st inst., two hundred and nineteen thousand six hundred and thirteen animals were slaughtered for food in New-York City. During the nine months of the year, the total number of animals offered up on the altar of appetite in that city, reached seven hundred thousand seven hundred and fourteen, or, at the rate of seventeen thousand nine hundred and sixty-six per week, or nearly one million per year! The population is about five hundred thousand—that is an average of two animals per year to each inhabitant. Not much starving in New-York, at this rate.—*Albany Register.*

---

Happy is the Wooing that is not Long Adoing.—An eminent writer says: "It is my firm opinion, derived from experience, that the period of courtship cannot be too short. I have reason to say, that when you have hooked your fish, the sooner you use your landing net the better."

---

To avoid Smoke.—It is said, that if a silk handkerchief is wetted and placed over the face, a person may pass through dense smoke without inconvenience.

A Fact for our Horticultural Readers.—It has been discovered that for the generality of flowers, and more especially, for geraniums, and the most delicate specimens of the lily tribe, *common glue*, diluted with a sufficient portion of water, forms a richer manure than guano or any other yet discovered. Plants placed in sand on the worst soils, display more beauty and vigor when watered with this composition, than those grown in the richest mould and only sprinkled with water.

---

A Family of Opium Lovers.—Some six months ago, a person visited our town, asking money to purchase medicines for his mother, who was sick. Recently, the same solicitor has been around on the same errand for other members of the family. What success has attended his solicitations, we know not; but the object to which the funds are applied, is of so objectionable a nature that all should withhold their names, out of regard for the family, who are the slaves of a habit to which drunkenness the most degrading is a comparative blessing. The entire family, it is said, subsist for the most part on opium, or its exhilarating and soporific influence, and this fearful habit has been so long indulged as to have grown into a second life. The example of Coleridge before the world, who acknowledged it the basest and most destructive of vices, and at the same time the most absolute of tyrants should, we think, be a sufficient warning to all after-comers to avoid the drug; but here is an example of a whole family addicted to the habit; and it has brought upon them all its certain results—apathy, indolence, poverty, misery—and will eventually end in the most wretched death.—*Elmira Republican.*

---

FAMILY JARS.

Jars of jelly, jars of jam,
Jars of pottled beef and ham,
Jars of early gooseberries nice,
Jars of mince meat, jars of spice,
Jars of orange marmalade,
Jars of pickles, all home-made,
Jars of cordial elder-wine,
Jars of honey, superfine;
Would that only jars like these,
Could be found in families!

Happy Marriages.—We clip the following official table from an English paper, giving a view of the connubial bliss in the city of London:

| | |
|---|---|
| Runaway wives | 1,132 |
| Runaway husbands | 2,348 |
| Married persons legally divorced | 4,175 |
| Living in open warfare | 17,345 |
| Living in private misunderstanding | 13,279 |
| Mutually indifferent | 55,340 |
| Regarded as happy | 3,175 |
| Nearly happy | 127 |
| Perfectly happy | 13 |

N. B. The above census was taken by an old bachelor.—Ed.

---

Secret of Comfort.—Though sometimes small evils, like invisible insects, inflict pains, and a small hair may stop a vast machine, yet the chief secret of comfort lies in not suffering trifles to vex one, and prudently cultivating an undergrowth of small pleasures, since very few great ones, alas! are let on long leases.—*Sharp's Essays.*

---

Delirium Tremens.—A boy, calling on a doctor to visit his father, who had delirium tremens, and not rightly recollecting the name of the disease, called it the *devil's trembles,* thus making bad Latin, but very good English.

---

Johnny Cakes.—Scald a quart of sifted Indian meal with sufficient water to make a thick batter; stir in a tablespoonful of salt; flour the hands well, and mould it into small cakes; fry them in fat enough nearly to cover them. When brown upon the under side they should be turned. It takes about twenty minutes to cook them. When done, split and butter them.

---

Fried Apples.—Wash them, cut in two, remove the stem and core, and unpealed put them into a pan with butter and some water, put on the lid, place them on a stove, stir them now and then until they are soft, but do not burn them. Sour apples do not fry well.

